# Cushing’s syndrome: a combined treatment with etomidate and osilodrostat in severe life-threatening hypercortisolemia

**DOI:** 10.1007/s42000-022-00397-4

**Published:** 2022-09-21

**Authors:** Lukasz Dzialach, Joanna Sobolewska, Wioleta Respondek, Agnieszka Wojciechowska-Luzniak, Przemyslaw Witek

**Affiliations:** 1 Department of Internal Medicine, Endocrinology and Diabetes, Medical University of Warsaw, Warsaw, Poland; 2 Department of Internal Medicine, Endocrinology and Diabetes, Mazovian Brodnowski Hospital, Warsaw, Poland

**Keywords:** Cortisol, Cushing’s syndrome, Etomidate, Osilodrostat, Severe hypercortisolemia

## Abstract

Endogenous Cushing’s syndrome (CS) is associated with increased morbidity and mortality. Early diagnosis and initiation of therapy are essential, but effective treatment remains a challenge. In a long-term follow-up, biochemical control of hypercortisolemia, especially when severe, is difficult to achieve. Life-threatening hypercortisolemia is difficult to control due to the limitations of pharmacotherapy, including its side effects, and may require etomidate infusion in the intensive care unit (ICU) to rapidly lower cortisol levels. The effectiveness of hypercortisolemia management can be increased by a dual blockade of cortisol production. We report the efficacy, safety, and tolerability of combined therapy with two steroidogenesis inhibitors, etomidate, and osilodrostat, in a 32-year-old woman diagnosed with severe ACTH-dependent hypercortisolemia, subsequently maintaining a stable level of cortisol with osilodrostat monotherapy. This approach enabled achievement of relatively rapid control of the hypercortisolemia while using an etomidate infusion and concomitant increasing doses of oral osilodrostat applying a “titrations strategy.” Our experience shows that it is worth taking advantage of the synergistic anticortisolic action of etomidate with osilodrostat.

## Introduction

Endogenous Cushing’s syndrome (CS), which results from excess cortisol production, has an annual incidence of 0.2 to 5 cases per million population [1, 2]. Approximately 80% of all endogenous CS cases are caused by corticotrophin (ACTH) hypersecretion (ACTH-dependent CS), of which 90% are represented by corticotroph pituitary adenomas (Cushing’s disease, CD), while ectopic ACTH syndrome (EAS) occurs in about 10% of cases [1, 2]. CS is associated with increased morbidity and mortality; thus, early diagnosis and implementation of therapy are necessary to prevent numerous complications, especially in the case of severe hypercortisolemia (SH) [3–5]. SH is defined as a significantly elevated random serum cortisol level (> 36–41 μg/dL), a 24-h urinary free cortisol (UFC) level greater than four-fold the upper limit, and/or profound hypokalemia (< 3.0 mmol/L) [6, 7].

The treatment of choice for CS, which gives the possibility of complete recovery, is surgical resection of the primary lesion which caused excess cortisol production [2, 8]. Pharmacological therapy is essential, especially in patients with contraindications to surgical procedure, as adjuvant treatment when the surgery was not radical, and in recurrent cases of CS [9]. Prompt pharmacological intervention leading to normalization of cortisol production is crucial in the case of life-threatening SH and while awaiting surgery (bridging therapy) since there might be a significant delay before an unequivocal diagnosis is established [6, 9]. Medical treatment options include steroidogenesis inhibitors, pituitary-targeting agents, and glucocorticoid receptor antagonists [8, 10]. These drugs can be used synergistically, increasing the potency of anticortisolic action and reducing the risk of possible side effects by lowering the dose of the individual agent [2]. The choice of a particular medication and therapeutic protocol is highly individualized and depends on the center’s experience; however, etomidate — a short-acting intravenous anesthetic agent — is considered the most effective for the rapid inhibition of cortisol overproduction [2, 10–12]. It acts by reversible blockage of the 11-β-hydroxylase enzyme (the final step of cortisol biosynthesis) and, additionally, 17α-hydroxylase/17,20-lyase and cholesterol side-chain cleavage enzyme [13–16]. Alternatively, rapid-acting oral steroidogenesis inhibitors, such as ketoconazole, metyrapone, or osilodrostat, might be considered in SH management. Osilodrostat is a novel adrenostatic agent that inhibits the activity of 11-β-hydroxylase as well as 18-hydroxylase enzyme (suppressing cortisol and also aldosterone synthesis) [17–21]. Recent reports show that osilodrostat can control SH relatively quickly [22–24].

A potential therapeutic option for life-threatening hypercortisolism is the use of combined treatment with etomidate and osilodrostat; however, no such procedure has to date been documented. Herein, we present a patient with ACTH-dependent SH brought under control with the combinative therapy of two steroidogenesis inhibitors, etomidate and osilodrostat.

## Case presentation

A 32-year-old female, diagnosed with multiple sclerosis (SM) years ago and treated with dimethyl fumarate for 2.5 years, was admitted to the Department of Internal Medicine, due to deterioration of her general condition, polydipsia, polyuria, qualitative disturbance of consciousness, and profound hypokalemia (1.9 mmol/L) found during SM therapy routine assessment. For about 2 months before the hospital admission, facial hair growth, acne lesions, rounded facial features without accompanying weight gain, and a tendency to bruise had been observed. In addition, at the same time, secondary amenorrhea was noted: the patient had been taking contraceptive drugs for 2 years and had discontinued them a few days after the onset of symptoms.

Physical examination revealed elevated blood pressure, moon face with visible acne lesions, and muscular atrophy of the limbs. BMI was 22.4 kg/m^2^. The laboratory tests showed severe hypokalemia (2.2 mmol/L), hyperglycemia meeting the criteria for diabetes mellitus with an HbA1c value of 5.9%, and preserved C-peptide secretion (3.5 ng/mL, *N*: 0.78–5.19). During the hospital stay, oral hypoglycemic drugs (linagliptin and empagliflozin) were administered. Despite compensation of disturbances in the water–mineral balance, it was not possible to stabilize the kalemia.

Due to the clinical features of hypercortisolemia, the hormonal diagnostics was extended: the abnormal circadian rhythm of cortisol secretion was confirmed and impaired glucocorticoid feedback was demonstrated with an overnight 1 mg dexamethasone suppression test. A significant degree of ACTH-dependent hypercortisolemia was diagnosed [UFC: 7155 μg/24 h (*N*: 4.3–176) midnight serum cortisol: 69.64 µg/dL (*N*: < 5.4), ACTH: 167 pg/mL (*N*: 6–48)].

During hospitalization in the Department of Internal Diseases, the psychotic symptoms worsened: the patient was consulted psychiatrically and treated with quetiapine, hydroxyzine, and lorazepam. Due to the exhaustion of diagnostic and therapeutic possibilities in the abovementioned hospital, the patient was referred to our Clinic — the Department of Internal Medicine, Endocrinology and Diabetes, Medical University of Warsaw, Warsaw, Poland — for treatment continuation.

**Fig. 1:**
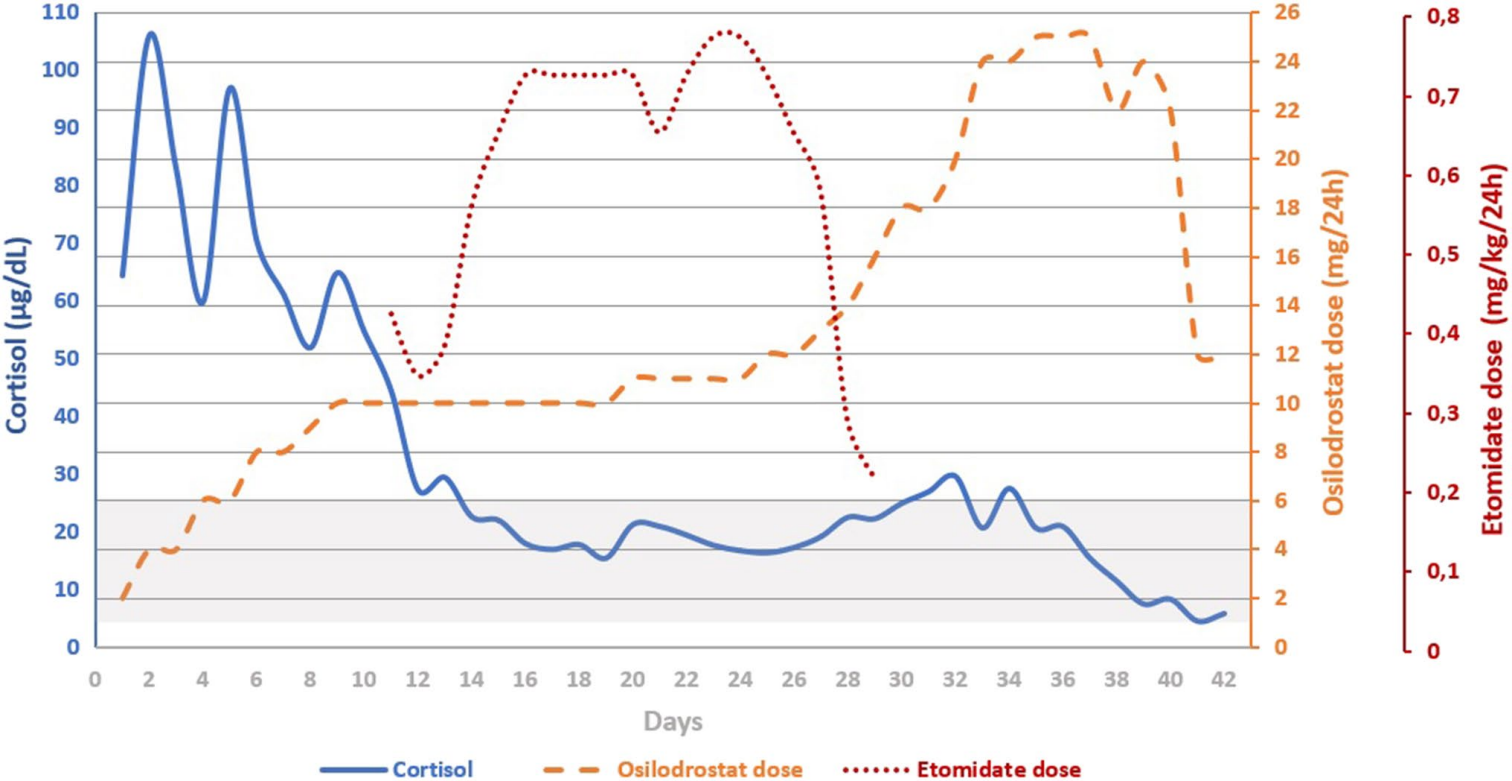
Evolution of serum cortisol levels during treatment with the osilodrostat and etomidate dosing regimen. Cortisol values are the average of 3–8/day time measurements (depending on the day). The shaded area corresponds to the cortisol reference range

Laboratory tests on admission to the Department of Endocrinology revealed profound hypokalemia (2.1 mmol/L) and elevated levels of serum cortisol (106 μg/dL), ACTH (154 pg/mL) and androgens [DHEA-S 1200 μg/dL (*N*: 95.8–511.7), and total testosterone 4.29 ng/dL (*N*: 0.38–1.97)]. The CRH stimulation test showed a significant (over fivefold) increase in concentration of ACTH and cortisol (by 33%). Treatment with an oral steroidogenesis inhibitor, osilodrostat, was started at increasing doses, which improved the patient’s general condition in the first days of therapy. Osilodrostat dose titration is presented in Fig. 1. During treatment, transient increase in alanine aminotransferase activity (122 U/L, *N*: 0–55) and persistent hypokalemia were observed despite intravenous and oral potassium supplementation and high doses of spironolactone.

**Fig. 2:**
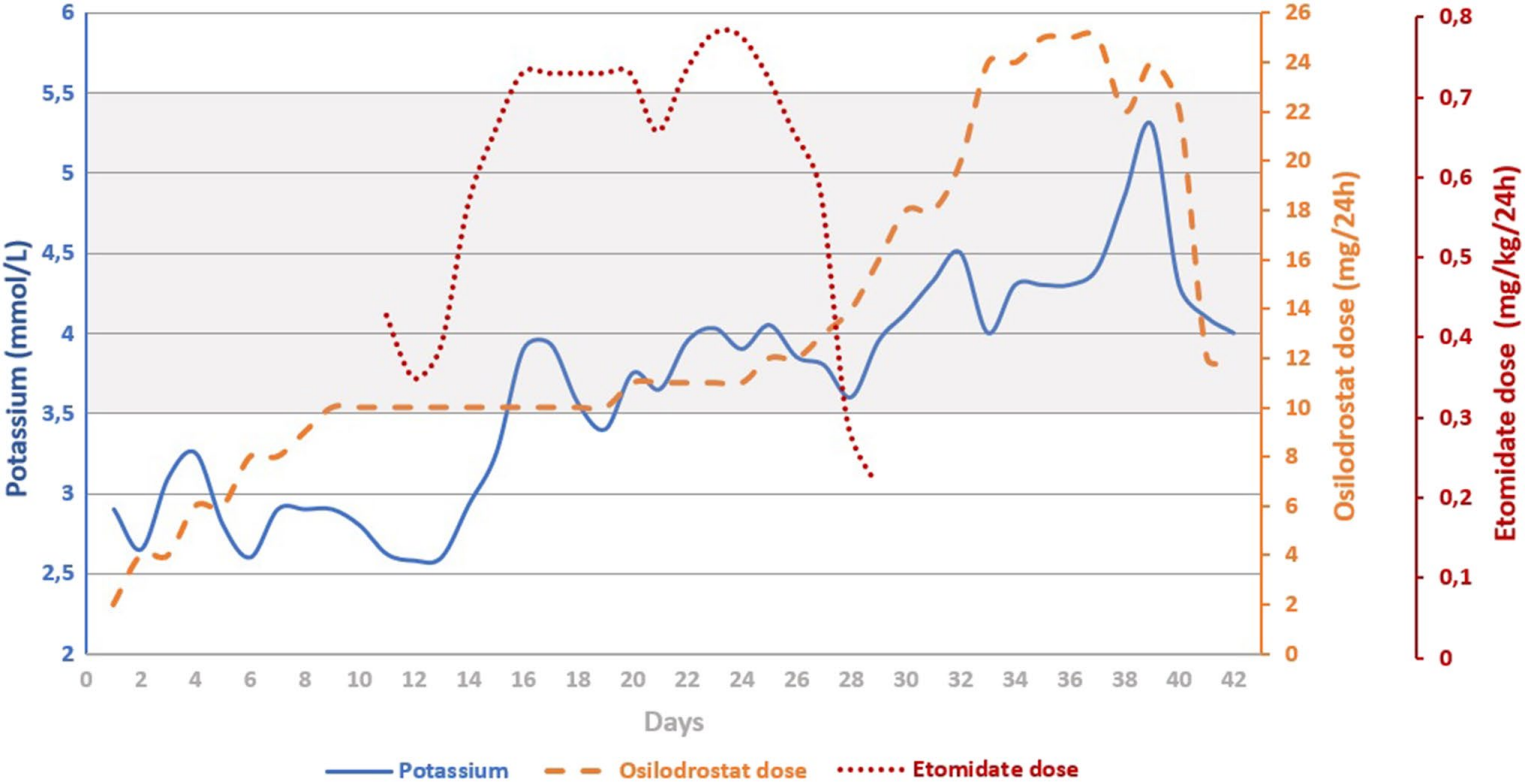
Evolution of potassium levels during treatment with the osilodrostat and etomidate dosing regimen. Potassium values are the mean of 1–8/day time measurements (depending on the day). The shaded area corresponds to the potassium reference range

Due to the ineffectiveness of the above treatment in lowering the serum cortisol, persistent hypokalemia (Fig. 2), increasing severe edema of the lower limbs found in the physical examination (weight gain by 3.1 kg within 7 days), and the patient’s reported significant deterioration in well-being, on the 11th day of treatment with osilodrostat, after obtaining the patient’s consent, it was decided to begin therapy with etomidate combined with the current cure with osilodrostat at a gradually increasing dose (Fig. 1). Etomidate therapy was conducted in the conditions of an intensive internist supervision room (non-ICU) at the initial dose 0.015 mg/kg/h and then modified according to the value of cortisolemia, kalemia, and glycemia and blood pressure control. During the treatment with etomidate, the monitoring of potassium and cortisol values was performed several times a day, including every 3 h on the first days of treatment. The patient tolerated the combined therapy (etomidate and osilodrostat) well and during the following days, an improvement in her general condition was observed, including mood balancing and reduction of lower limb swelling. Blood pressure and blood glucose control were correct. Nausea and vomiting were not observed. The patient reported persistent dizziness, which she also experienced after completing the etomidate infusion. During treatment with etomidate, the patient obtained from + 1 to 0 points on the RASS scale. In the first 2 days of combined treatment, due to increased hypokalemia (Fig. 2), the patient required intravenous and oral potassium substitution, and then only oral substitution in combination with spironolactone, which was part of the antihypertensive treatment.

After stabilization of cortisol levels and kalemia, treatment with etomidate was discontinued on the 19th day of the combined therapy (on the 29th day of osilodrostat treatment), and treatment with an oral steroidogenesis inhibitor was continued at doses modified according to the performed hormonal assessments. On the following days, a decrease in the requirement for osilodrostat was observed. In the course of the treatment, the activity of ALT was also normalized, and the levels of testosterone (2.96 nmol/L) and DHEA-S (185 μg/dL) decreased pronouncedly.

Due to the significant drops in blood pressure, the antihypertensives were firstly reduced and then discontinued and the correct values were obtained in the control measurements. During hospitalization at the clinic, the patient was consulted once again psychiatrically: the quetiapine treatment was supplemented with pregabalin, the dose of which was reduced as recommended after the correction of cortisol level.

**Fig. 3:**
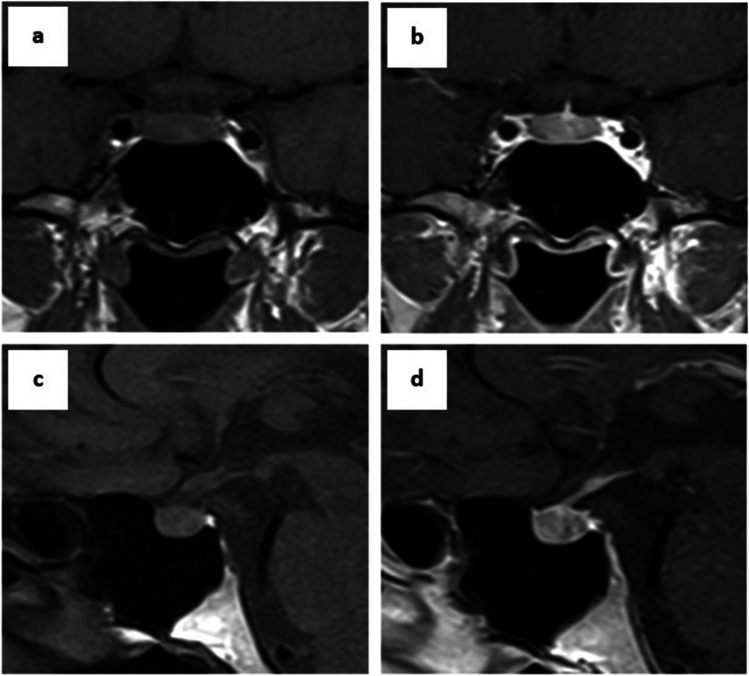
Frontal and sagittal MRI demonstrating a normal pituitary without signs of microadenoma in the presented patient: **a** Frontal T1-weighted pre-contrast scan. **b** Frontal T1-weighted post-contrast scan. **c** Sagittal T1-weighted pre-contrast scan. **d** Sagittal T1-weighted post-contrast scan

To further diagnose ACTH-dependent hypercortisolemia, an MRI of the pituitary gland was performed in which the inconclusive image as to the presence of microadenoma was visualized (Fig. 3). In the opinion of the experienced consultant neurosurgeon, an ambiguous change in the pituitary gland is not the source of the patient’s autonomous production of ACTH. To search for the ectopic source of ACTH secretion, a chest-abdomen-pelvis CT with contrast was performed in which no abnormalities were visible, while positron emission tomography (PET) with ^68^ Ga-DOTA-conjugated peptides showed no areas of increased tracer accumulation.

The patient was discharged from the clinic in good general condition, with the final cortisol levels as shown in Fig. 1. Continuation of the treatment with osilodrostat at a dose of 6 mg twice a day, quetiapine, pregabalin, and prolonged-release metformin was recommended. A follow-up took place after 14 days. The patient was in good general condition and did not report any complaints. Morning serum cortisol (14.3 µg/dL), UFC (103 µg/24 h), DHEA-S (104.3 μg/dL), and potassium level (4.0 mmol/L) were within normal range. An increased level of testosterone was observed (6.06 nmol/L). The dose of osilodrostat was maintained. The patient is still currently followed up at our clinic.

## Discussion

The treatment of CS is complex and challenging as no medication has complete and definitive efficacy in hypercortisolemia management. A large, multicenter study showed that biochemical control is not achieved in up to 30% of patients with CD in a long-term follow-up [25]. Conventional pharmacological therapy is often ineffective in controlling life-threatening SH. The use of the drugs individually is also limited by the frequent occurrence of their side effects. A possible therapeutic approach, which minimizes adverse events and increases treatment efficacy, is to use two or more drugs simultaneously with an additive synergistic or/and complementary mechanism of anticortisolic action. The most common is the combined use of high doses of metyrapone and ketoconazole [26–28]. There are also data presenting the option of a triple combination with the additional usage of mitotane [29]. Despite good effectiveness, the latter treatment protocols were, however, associated with numerous serious side effects (mainly liver damage) [27, 29, 30]. In SH, the use of mitotane is limited due to its relatively slow onset of action [29]. Steroidogenesis inhibitors have also been studied in combination with pituitary-directed agents (pasireotide and cabergoline); however, such therapy is used in patients with persistent or recurrent CD rather than for the purpose of controlling SH [31–34]. Lawrence et al. reported a patient with severe EAS, due to a thymic neuroendocrine tumor, who was successfully controlled with a combination regimen of ketoconazole and mifepristone [35]. However, it is difficult to monitor the efficacy and safety of such treatment because measuring serum cortisol and/or UFC is not objective in that case [36].

Osilodrostat is a particularly potent oral and selective inhibitor of 11-beta-hydroxylase compared with other steroidogenesis inhibitors [19]. It also has a prolonged half-life compared with metyrapone, enabling less frequent medication intake, which may improve patient compliance [17, 19]. Typically, in CD, it is initiated at 2 mg orally twice daily and titrated by 1–2 mg every 2 weeks based on cortisol level [8]. In a phase III study (LINC 4) in patients with CD, osilodrostat normalized UFC excretion in 77% of patients after 12 weeks’ treatment [20]. Recently, osilodrostat has also been shown to have the potential of controlling SH relatively quickly; however, limited relevant data are available [22, 23]. Haissaguerre et al. reported three patients with SH treated successfully with osilodrostat monotherapy [22]. Starting doses (2–7 mg/day) were titrated every 2–5 days (reaching maximal doses of 7–44 mg/day) and normal plasma cortisol concentration was achieved within 2 weeks [22]. Subsequently, Bessiène et al. reported a case of severe EAS and presented a regimen with an even higher starting dose of osilodrostat (20 mg/day) applying the “block and replace” strategy [23]. This approach allowed for the normalization of cortisol levels in only 6 days [23]. Recently, Amodru et al. also reported successful hypercortisolism control with ketoconazole-osilodrostat combination therapy [24].

In the presented patient, osilodrostat was introduced at 2 mg BID and the dose was gradually increased on subsequent days using a dose-titration strategy. The serum cortisol level was more than halved after 6 days of treatment. On the 10th day, the patient reported worsening well-being, and an increase in peripheral edema was observed. Morning cortisol at that time was 62.9 µg/dL. Due to the deterioration of the patient’s condition, insufficient response to current treatment, and no clear protocol for the osilodrostat monotherapy in SH, the patient was started on a continuous etomidate infusion. We decided to continue osilodrostat simultaneously and, therefore, the patient did not receive the initial bolus of etomidate, while the starting dose of medication (0.015 mg/kg/h) was lower than that assumed in most treatment protocols [2, 11, 37, 38]. The premise of that approach was to gradually reduce cortisolemia through combination therapy, with cross-titration of osilodrostat and etomidate so that the etomidate infusion could be stopped with the serum cortisol in the desired range and maintained through osilodrostat monotherapy.

The tolerance of combined osilodrostat and etomidate therapy was surprisingly good: apart from persistent dizziness, the patient did not report any additional side effects. Mild ALT elevation was noted after starting osilodrostat, but it normalized spontaneously within further hospitalization. During etomidate infusion, the patient was assessed with the RASS scale, obtaining + 1 point on the starting day of the combinative treatment and 0 points on each subsequent day until the end of the infusion. However, the patient’s condition during the first day of etomidate therapy reflected the effect of hypercortisolemia rather than the side effect of etomidate initiation. This is also evidenced by the fact that despite increasing the dose of etomidate, the patient’s assessment on the RASS scale did not change. Moreover, studies suggest that sedative symptoms of etomidate are not experienced at doses smaller than 0.1–0.3 mg/kg/h [39].

The combined therapy lasted 19 days and etomidate infusion was discontinued at the 18 mg daily osilodrostat dose. At that time, serum cortisol and UFC were 17.7 µg/dL and 243 µg/24 h, respectively. Unfortunately, UFC rebounded in 48 h to 3493 µg/24 h and, therefore, the osilodrostat dose was increased to 25 mg/day over the next few days. Unexpectedly, over the following days, a marked decline in cortisol serum level (4.4 µg/dL) and UFC (12 µg/24 h) was noted, and the osilodrostat dose was reduced by half. The patient was discharged home in good general condition on a stable dose of osilodrostat (12 mg/day). After 14 days, at the follow-up, morning serum cortisol (14.3 µg/dL), UFC (103 µg/24 h) and potassium level (4.0 mmol/L) were correct and osilodrostat dose remained unchanged.

There was also a notable decrease in testosterone and DHEA-S levels during the hospitalization. An increase in testosterone concentration may be expected while using osilodrostat because of compensatory ACTH stimulation and shift from corticosteroid to androgen synthesis [19, 40]. Probably, this concerned the etomidate effect on the activity of other adrenal steroidogenesis enzymes [15, 16]. Some data suggest that higher doses of osilodrostat may affect the 17α-hydroxylase/17,20-lyase activity leading to decreased adrenal androgens synthesis [19, 40]. However, at the follow-up, testosterone level notably increased (6.06 nmol/L), most likely as an osilodrostat side effect in the mechanism described above.

Possibly, the starting dose of osilodrostat in the presented patient could have been higher, with more rapid dose titration: that is, either we should have introduced etomidate earlier or increased its flow more quickly. However, that would have required the “block and replace” strategy, which should be reserved for the most severe and life-threatening cases of hypercortisolemia as in the previously mentioned case reported by Bessiène et al. [2, 23]. In addition, there are no supporting data on the superiority of such a regimen over the “titration strategy” in less severe cases. Furthermore, in the days following the combined treatment, we observed an improvement in the general condition of the patient, which reflected a gradual improvement in hypercortisolemia. Despite the longer time of hypercortisolemia normalization through combination therapy, we were able to balance the level of cortisol with the minimum risk of adrenal insufficiency in non-ICU conditions. Slower hypercortisolemia control also allows the patient to adjust to lower levels of cortisol and to let the receptors regain normal sensitivity [7].

## Conclusions

In conclusion, combined therapy with osilodrostat and etomidate was well tolerated and highly effective in controlling ACTH-dependent SH and provides an alternative to the current therapeutic regimens. This strategy enabled us to reduce the risk of medication side effects and control the level of cortisol with minimized risk of adrenal insufficiency in non-ICU conditions, despite the longer time of hypercortisolemia normalization. Complementary observations are needed to confirm the efficacy and safety of the above therapeutic approach in SH management and to establish optimal starting doses and cross-titration strategy of osilodrostat and etomidate.
